# The value of radial endobronchial ultrasound-guided bronchial brushing in peripheral non-squamous non-small cell lung cancer

**DOI:** 10.1038/s41598-018-24300-7

**Published:** 2018-04-11

**Authors:** Kai-Lun Yu, Tzu-Hsiu Tsai, Chao-Chi Ho, Wei-Yu Liao, Ching-Kai Lin, Chia-Lin Hsu, Jin-Yuan Shih

**Affiliations:** 1 Department of Internal Medicine, National Taiwan University Hospital Hsin-Chu Branch, No. 25, Ln. 442, Sec. 1, Jingguo Road, North Dist, Hsinchu City, 30059 Taiwan; 2 Graduate Institute of Clinical Medicine, College of Medicine, National Taiwan University, No. 7 ChungShan South Road, Taipei, 10002 Taiwan; 3 Department of Internal Medicine, National Taiwan University Hospital, No. 7 ChungShan South Road, Taipei, 10002 Taiwan

## Abstract

Radial endobronchial ultrasound (R-EBUS) is one important diagnostic approach in non-small cell lung cancers (NSCLC). However, the small samples obtained from R-EBUS-guided transbronchial biopsies are sometimes insufficient for pathological and molecular diagnosis. Herein, we investigated the suitability of R-EBUS-guided bronchial brushing specimens for NSCLC diagnosis and EGFR genotyping. We enrolled 941 consecutive patients with peripheral pulmonary lesions who underwent R-EBUS. Cytology-positive brushing specimens from non-squamous NSCLC patients were tested for EGFR mutations. Non-squamous NSCLC was diagnosed in 624 patients (66.3%). Positive cytology was documented in the brushing samples of 376 patients (60.3%). Higher diagnostic yields were obtained in patients exhibiting bronchus signs on chest tomography, and those with R-EBUS probe located within the lesion. EGFR genotyping was successfully performed in 363 samples (96.5% of cytology-positive brushing samples). EGFR genotyping concordance between brushing specimens and matched tissue samples was 88.7% (kappa = 0.745, P < 0.001). Furthermore, 144 non-squamous NSCLC patients (23.1%) with failed pathological diagnosis or EGER sequencing by R-EBUS-guided transbronchial biopsy required repeat biopsies. However, it was achieved successfully from the brushing specimens of 57 patients (39.6%). In conclusion, for patients with peripheral lung cancer, R-EBUS-guided bronchial brushing could provide an additional sampling method for diagnosis and EGFR genotyping.

## Introduction

The discovery of mutations in the epidermal growth factor receptor (*EGFR*), as well as that of EGFR-tyrosine kinase inhibitors (TKIs), introduced a new era of precision medicine in lung cancer treatment^1,2^. Clinical studies demonstrated the significant treatment efficacy of targeted therapy in patients with sensitizing *EGFR* mutations^3–5^. As a result, current guidelines for the diagnosis and treatment of patients with advanced non-small cell lung cancer (NSCLC) recommend that *EGFR* genotyping be performed in tumors with non-squamous histologies^6,7^.

In many patients with NSCLC, especially those with tumors of non-squamous histologies, tumors often present with peripheral pulmonary lesions (PPLs) that impede access to the target tumor by conventional bronchoscopy. Indeed, these lesions present a frequently encountered challenge to pulmonologists. With the development of radial endobronchial ultrasound (R-EBUS), which dramatically improved the visualization and localization of PPLs, the diagnostic yield of transbronchial biopsy has improved^8,9^. Adequate biopsy tissue samples are required for the diagnosis, subtyping, and genotyping of NSCLC samples. However, specimens obtained via transbronchial biopsy are often small and contain a limited number of tumor cells, precluding further molecular testing^10–12^. In such cases, patients require repeat procedures, such as computed tomography (CT)-guided transthoracic needle biopsy or surgical resection, to obtain additional tissue samples for molecular testing. Hence, patients undergoing repeat invasive procedures are exposed to additional risks, and treatment may be delayed as a result.

Our previous pilot study showed that RNA-based sequencing of waste bronchial brushing specimens may be a feasible method for multi-gene analysis^13^. However, no cohort studies have been performed to investigate the role of R-EBUS-guided bronchial brushing in cytopathological diagnosis and *EGFR* analysis. Therefore, in the present study, we investigated the performance of R-EBUS-guided bronchial brushing in both the cytopathological diagnosis of and *EGFR* mutation detection in peripheral non-squamous NSCLC.

## Methods

### Study design and settings

This study was conducted at National Taiwan University Hospital, a tertiary referral center. Consecutive patients with PPLs who were referred for R-EBUS between September 2010 and December 2015 were enrolled (n = 941). A PPL was defined as a lesion surrounded by lung parenchyma with no endobronchial abnormalities detected by conventional bronchoscopy. Computed tomography-based findings, including tumor location, size, and presence of a bronchus sign (i.e., a bronchus leading directly to a PPL) were documented. The treatment responses of patients with advanced NSCLC who were administered first-line EGFR-TKIs (erlotinib, gefitinib, or afatinib) were recorded based on the Response Evaluation Criteria in Solid Tumors, version 1.1^14^. The cutoff date for data collection was November 31, 2016. This study was approved by the Institutional Review Board of National Taiwan University Hospital. Written informed consent was obtained from all patients before undergoing bronchoscopic procedures. All methods were performed in accordance with the relevant guidelines and regulations.

### EBUS-guided procedures

Conventional bronchoscopy (BF-P260F or BF-P290; Olympus, Tokyo, Japan) was initially performed to examine the trachea and bronchi. R-EBUS was then performed using an endoscopic ultrasound center (EU-M30S; Olympus) and a 20-MHz radial ultrasonic probe (UM-S20-20R; Olympus). The R-EBUS probe position was recorded as within or adjacent to the target tumor. After a lesion was located, the radial probe was withdrawn from the working channel of the bronchoscope, and the R-EBUS procedure consisting of transbronchial biopsy, bronchial brushing, and bronchial washing was then performed.

### Specimen preparation

Each specimen obtained by bronchial brushing was first smeared onto slides. Air-dried smears and those fixed in 95% ethyl alcohol were prepared for routine evaluation. Next, the brushing head was removed and dipped into TRI reagent solution (Molecular Research Center, Cincinnati, OH), and specimens were stored as described previously^15^. The bronchial brushing cytology slides were examined by a board-certified cytopathologist. *EGFR* mutation analysis was then performed on tumor cell-containing bronchial brushing specimens obtained from patients diagnosed with non-squamous NSCLC.

### *EGFR* mutation analysis of brushing specimens

Reverse transcription-polymerase chain reaction (RT-PCR) on RNA extracted from the brushing specimens was performed with the Qiagen One-Step RT-PCR Kit (Qiagen, Hilden, Germany) using previously reported conditions and primers^13,16^. The RT-PCR amplicons were purified and sequenced with a BigDye Terminator Sequencing Kit (Applied Biosystems, Foster City, CA). Sequencing products underwent electrophoresis on an automated ABI PRISM 3700 genetic analyzer (Applied Biosystems). Both the forward and reverse sequences obtained were analyzed, and chromatograms were examined manually.

### *EGFR* mutation analysis from matched histological specimens

Matched histological specimens, including biopsy specimens and surgical tissues, were used for *EGFR* mutation detection. *EGFR* mutations were analyzed using standard methods at our institution, including either direct sequencing or matrix-assisted laser desorption ionization-time of flight mass spectrometry (MALDI-TOF MS), as previously described^13,17–19^.

### Statistical analysis

For univariate analysis, categorical variables were analyzed using the chi-squared test or Fisher’s exact test as appropriate. For multivariate analysis, variables with P-values of <0.10 were incorporated into the multivariate logistic regression model to identify independent factors. Intra-individual agreements between the different methods used to detect *EGFR* mutations were determined by calculating Cohen’s kappa coefficient. Progression-free survivals after treatment with EGFR-TKIs were analyzed using the Kaplan-Meier method. All analyses were conducted using SPSS for Windows, version 15.0 (SPSS Inc., Chicago, IL, US). A two-tailed P-value of <0.05 was considered statistically significant.

## Results

### Patient characteristics

**Figure 1:**
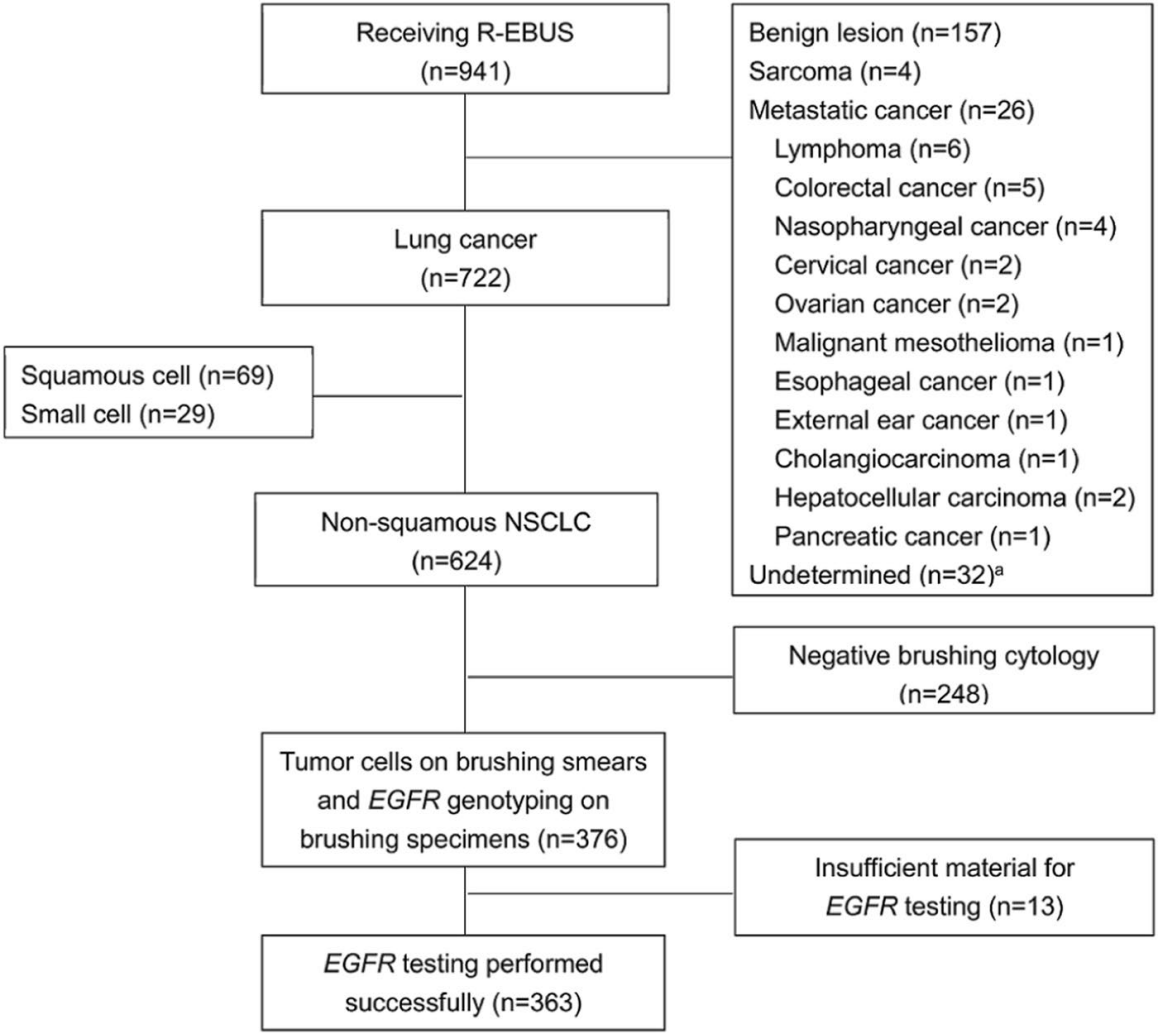
Study schematic^a^. Thirty-two patients remained undiagnosed after bronchoscopic examination and were lost to follow-up. Radial-EBUS, radial endobronchial ultrasound; NSCLC, non-small cell lung cancer.

A total of 941 consecutive patients who underwent R-EBUS were enrolled in this study. The final diagnoses of these patients, established either by R-EBUS or other diagnostic procedures, are shown in Fig. 1. Among these patients, 722 were diagnosed with lung cancer, 624 of whom (86.4%) had non-squamous NSCLC; the clinical features of the latter group of patients are shown in Table 1. R-EBUS-guided transbronchial biopsy and bronchial brushing cytology established the cytopathological diagnosis of 427 (68.4%) and 376 (60.3%) non-squamous NSCLC patients, respectively. Non-squamous NSCLC was identified in 489 patients (78.4%) when the results of both transbronchial biopsy and bronchial brushing cytology were considered together, while 62 patients (9.9%) were diagnosed solely on the basis of bronchial brushing cytology. Samples from the 376 patients with tumor cells found on their brushing smears underwent RT-PCR and Sanger sequencing for *EGFR* mutation analysis.

**Table 1:** Clinical characteristics of 624 non-squamous NSCLC patients receiving R-EBUS.

Variable	Subsets	Number	(%)
Age, years	Mean	65.2	
	Range	18–95	
Sex	Male	292	(46.8)
	Female	332	(53.2)
Smoking history	Never	425	(68.1)
	Former/current	199	(31.9)
Tumor type	Adenocarcinoma	575	(92.1)
	NSCLC, not specified	26	(4.2)
	NSCLC, other cell types^a^	23	(3.7)
PS^b^	0–1	549	(88.0)
	≥2	75	(12.0)
Stage^c^	IA	43	(6.9)
	IB	56	(9.0)
	IIA	24	(3.8)
	IIB	9	(1.4)
	IIIA	70	(11.2)
	IIIB	29	(4.6)
	IV	390	(62.5)
	N/A	3	(0.5)
Lobe	Left upper lobe	149	(23.9)
	Left lower lobe	97	(15.5)
	Right upper lobe	189	(30.3)
	Right middle lobe	88	(14.1)
	Right lower lobe	101	(16.2)

NSCLC, non-small cell lung cancer; R-EBUS, radial endobronchial ultrasound; PS, performance status; N/A, not available.

^a^Includes adenosquamous carcinoma (n = 6), pleomorphic carcinoma (n = 3), lymphoepithelioma-like carcinoma (n = 6), sarcomatoid carcinoma (n = 4), neuroendocrine carcinoma (n = 1), large cell carcinoma (n = 1), adenocarcinoma with small cell component (n = 2).

^b^Based on the Eastern Cooperative Oncology Group performance status score.

^c^Based on the 7^th^ edition of the TNM (tumor, node, and metastasis) classification of lung cancer.

### Predictive factors for positive brushing cytology results

Factors associated with the diagnostic yield of non-squamous NSCLC samples are shown in Table 2. In univariate analysis, the diagnostic yield of brushing samples was significantly associated with tumor size and presence of a bronchus sign on chest CT, and R-EBUS probe position. Multivariate analysis showed that significantly higher diagnostic yields were associated with a bronchus sign and the localization of the R-EBUS probe located within the lesion (P < 0.01, odds ratio [OR]: 1.97, confidence interval [CI]: 1.39–2.80; P < 0.01, OR: 2.37, 95% CI: 1.56–3.60, respectively).

**Table 2:** Predictive factors of diagnostic yield by R-EBUS-guided bronchial brushing in 624 non-squamous NSCLC patients.

Variables	Diagnostic yield (%)	Univariate analysis		Multivariate analysis	
		OR [95% CI]	P-value	OR [95% CI]	P-value
Total	376/624 (60.3)				
Sex					
Male	181/292 (68.1)	1.15 [0.83–1.58]	0.41		
Female	195/332 (58.7)				
Smoking					
Smoker	110/186 (59.1)	0.97 [0.81–1.15]	0.71		
Non-smoker	266/438 (60.7)				
Performance status score					
≥2	40/75 (53.3)	0.73 [0.45–1.18]	0.20		
<2	336/549 (61.2)				
Tumor size					
≥2 cm	354/576 (61.4)	1.97 [1.04–3.41]	0.04	1.77 [0.85–2.90]	0.15
<2 cm	22/48 (45.8)				
Lobar location			0.256		
Upper	209/338 (61.8)				
Middle	60/88 (68.2)				
Lower	107/198 (54.0)				
Bronchus sign					
Positive	281/432 (65.0)	1.90 [1.35–2.68]	<0.01	1.97 [1.39–2.80]	<0.01
Negative	95/192 (49.5)				
R-EBUS probe position					
Within the lesion	325/506 (64.2)	2.36 [1.57–3.54]	<0.01	2.37 [1.56–3.60]	<0.01
Adjacent/invisible the lesion	51/118 (43.2)				

R-EBUS, radial endobronchial ultrasound; NSCLC, non-small cell lung cancer; OR, odds ratio; CI, confidence interval.

### *EGFR* mutation analysis of samples obtained by R-EBUS brushing

Successful *EGFR* genotyping was achieved in bronchial brushing samples from 363 of 376 patients (96.5%), while samples from 13 patients (3.5%) failed *EGFR* amplification and sequencing because of insufficient material. *EGFR* mutations were detected in 216 patient samples; among them, 99 (38.5%) were exon 19 deletions, 84 (23.9%) were L858R, and 33 (9.4%) were other uncommon mutations.

### Comparison among *EGFR* analysis methods

Of the 363 patients for whom *EGFR* genotyping of brushing specimens was successful, 284 had matched samples available for *EGFR* testing using standard testing methods. Specifically, 204 specimens were genotyped using MALDI-TOF-MS, whereas 80 were analyzed with Sanger sequencing. Comparison of *EGFR* testing results between the bronchial brushing and matched histological samples (Table 3) yielded a concordance rate of 88.7%, with a kappa value of 0.745 (P < 0.001).

**Table 3:** Comparison of *EGFR* genotyping results between R-EBUS-guided bronchial brushing and patient-matched histological samples.

Matched samples	R-EBUS brushing cytology samples					
	Wild-type	Exon 19 del	L858R	Mixed	Others	Total
Wild-type	81	5	4	1	5	96
Exon 19 del	4	75	0	1	1	81
L858R	12	0	63	5	0	81
Mixed	3	2	3	4	0	12
Other	2	0	1	2	9	14
Total	104	83	71	13	15	284

R-EBUS, radial endobronchial ultrasound; del, deletion

Data are presented as numbers of samples.

### Performance of R-EBUS-guided bronchial brushing in patients with failed transbronchial biopsies

Of the 624 patients with non-squamous NSCLC who underwent R-EBUS transbronchial biopsy, 144 (23.1%) required repeat biopsies for pathological diagnosis or *EGFR* testing because of inaccessible transbronchial lesions (n = 6), negative transbronchial biopsy results (n = 107), or inadequate material (n = 31). Among patients requiring repeat biopsies, the R-EBUS brushing specimens of 57 patients (39.6%) provided positive cytology and successful *EGFR* sequencing results (Table 4). Fifteen patients with initial failed transbronchial biopsies underwent repeat R-EBUS-guided procedures, including transbronchial biopsy and bronchial brushing. Pathological diagnosis and EGFR genotyping were successfully performed with repeat R-EBUS-guided biopsies in 8 (53.3%) patients.

**Table 4:** R-EBUS bronchial brushing potentially prevented repeat biopsies in patients with failed transbronchial biopsy.

Repeat Procedures (n)	Successful bronchial brushing (n)
Repeat R-EBUS (15)	6
EBUS-TBNA (3)	1
US-guided TTB (18)	7
CT-guided TTB (53)	21
US-guided biopsy of neck LAP (9)	6
US-guided biopsy of axillary LAP (5)	2
Excision biopsy of neck LAP (6)	2
Thoracentesis (24)	8
Pleural biopsy (13)	1
Surgical resection^a^ (8)	3
Total (144)	57

R-EBUS, radial endobronchial ultrasound; EBUS-TBNA, endobronchial ultrasound-transbronchial needle aspiration; CT, computed tomography; TTB, transthoracic biopsy.

^a^Includes wedge resection (n = 6), medianoscope (n = 1), pericardiectomy (n = 1).

### Treatment efficacy of EGFR-TKIs according to *EGFR* mutation status of R-EBUS brushing specimens

Of the 419 patients with advanced (stage IIIB/IV) non-squamous NSCLC enrolled in this study, *EGFR* mutation profiles were obtained from the bronchial brushing specimens of 142 cases. Tumors from 121 patients harbored *EGFR* mutations, whereas 21 possessed wild-type *EGFR*. All patients for whom tumor *EGFR* mutation status was obtained were then treated with first-line EGFR-TKIs. Among these patients, those with *EGFR* mutations exhibited improved disease response (63.0% vs. 33.3%, P < 0.001) and disease control rates (95.0% vs. 5%, P < 0.001), as well as prolonged progression-free survival (11.7 months vs. 7.2 months, P = 0.006), compared to those without *EGFR* mutations.

## Discussion

Our findings support a role for R-EBUS-guided bronchial brushing samples in the cytological diagnosis and *EGFR* mutation analysis in patients with peripheral non-squamous NSCLC, thus allowing patients to avoid more invasive procedures.

Several previous studies have demonstrated the importance of *EGFR* mutation analysis in predicting the treatment efficacy of EGFR-TKIs in patients with NSCLC^1,3–5^. The reliable harvesting of samples for pathological diagnosis and *EGFR* testing is critical for patients with advanced non-squamous NSCLC. Marked progress in diagnostic bronchoscopy has been achieved in the last decade, and R-EBUS is one of the most important of these advances^20^. Previous studies have demonstrated that EBUS-guided bronchial brushing improved the diagnostic yields of samples from patients with PPLs^8,20–22^. However, few studies have investigated whether R-EBUS specimens are conducive for molecular diagnostics. Guiser *et al*. demonstrated that the molecular diagnosis of R-EBUS specimens was feasible for approximately 80% of patients with peripheral lung cancer^23^. However, the majority of these specimens were biopsy, rather than bronchial brushing, specimens, and the results from *EGFR* mutation analyses of brushing specimens were not compared with those from patient-matched histological samples.

Previous studies have investigated the utility of cytological specimens for molecular testing, but focused on fine-needle aspiration and pleural effusion analysis rather than brushing cytology specimens^24–26^. Our previous pilot study demonstrated the potential utility of EBUS-guided brushing specimens in molecular diagnostics^13^. However, the factors that predict diagnostic yields of R-EBUS-guided bronchial brushing samples were not assessed because of the relatively small population size. Therefore, in the current study, we investigated the feasibility of using R-EBUS-guided brushing specimens for the cytological diagnosis and *EGFR* mutation analysis of tumors in patients with peripheral non-squamous NSCLC.

In the present study, the utility of R-EBUS brushing samples for molecular diagnostics was determined. We observed that the diagnostic yields of specimens from R-EBUS-guided transbronchial biopsy, brushing cytology, and both techniques combined were 68.4%, 60.3%, and 78.4%, respectively, consistent with the results of previous studies^8,20,21^. Moreover, we found that the presence of a bronchus sign on chest CT, as well as the localization of the R-EBUS probe within the lesion, were independent factors that improved the diagnostic yield of R-EBUS-guided bronchial brushing specimens. Previous studies have shown that the location of the tumors may influence the diagnostic yield of R-EBUS-guided transbronchial biopsies^9,27,28^. To our knowledge, our study is the first to investigate the factors that predict the diagnostic yield of R-EBUS-guided bronchial brushing specimens.

*EGFR* mutation detection was performed successfully in 96.5% of the 363 R-EBUS-guided brushing specimens that contained tumor cells. We observed good concordance between the *EGFR* mutation status of brushing-derived vs. patient-matched histological specimens (kappa = 0.745). Using RT-PCR, we also identified rare mutations that were not detected with standard DNA-based methods^29^. Moreover, 142 advanced NSCLC patients with *EGFR*-mutant brushing specimens exhibited better treatment responses and progression-free survival compared to patients with *EGFR*-wild-type specimens. The treatment efficacy of EGFR-TKIs was similar to that observed in previous studies^3–5^.

Some discrepancies between the *EGFR* mutational profiles of the brushing samples and matched biopsy/resection specimens were noted. First, some patients with *EGFR*-wild-type biopsy specimens harbored *EGFR* mutations in their R-EBUS brushing specimens. The sensitivity of mutation assessment may be affected by interference from non-tumor cells in the biopsy specimens. Using an RT-PCR-based system for mutation detection may overcome discrepancies caused by heterogeneous specimens^15^. Second, some patients with a single mutation in their biopsy specimens harbored uncommon, complex mutations in their R-EBUS brushing specimens. The RT-PCR-based method with Sanger sequencing identified these uncommon, complex mutations, which is important for predicting the treatment efficacy of EGFR-TKIs^30^. Third, some *EGFR* mutations detected in biopsy specimens were not detected in the corresponding R-EBUS brushing specimens, potentially due to the limited number of tumor cells in the latter sample type. Moreover, tumor spatial heterogeneity may also lead to discrepancies in *EGFR* mutation status^31,32^.

In this study, 144 patients required repeat invasive procedures because of negative biopsy results or insufficient samples for EGFR mutation analysis. Among them, 76 patients (52.8%) had stage IV non-squamous NSCLC with various distant metastases, including pleura, pericardium, bone, brain, liver, adrenal metastases, and so on. Because of tumor size and location, some of these metastases were more difficult and invasive to approach compared with R-EBUS. In this context, acquisition of R-EBUS-guided bronchial brushing samples and the associated increased diagnostic yields facilitated *EGFR* mutation analysis. The simultaneous use of both techniques enabled successful pathological diagnosis and *EGFR* genotyping following a single procedure, averting the need for additional invasive procedures.

Advances in the molecular diagnosis of lung cancer include “liquid biopsy”, by which genotyping is performed on circulating tumor DNA in the plasma^33–37^; however, recent studies showed variable reliability and sensitivity when detecting mutations using this method; cost and technical problems were also considerations. Cytological/histological specimens and liquid biopsy may be considered as complementary methods of molecular testing.

Our results should be interpreted in light of the limitations of our study. First, the matched specimens for comparing *EGFR* mutation status were not obtained exclusively from R-EBUS transbronchial biopsies. Second, the study was conducted at a single institution; multi-center studies remain under consideration. Third, testing for other mutations, such as *ALK* translocation, was not performed in this study and should be investigated in further work. Finally, advances in bronchoscopy techniques, including guide sheath and electromagnetic navigation bronchoscopy, can increase sample diagnostic yield without causing additional complications, such as pneumothorax^38–40^. As such, the utility of these techniques to molecular diagnostics, together with R-EBUS-guided bronchial brushing, requires further investigation.

### Data availability

The datasets that were generated and/or analyzed during the current study are available from the corresponding author on reasonable request.
